# Research Progress on the Relationship between Atherosclerosis and Inflammation

**DOI:** 10.3390/biom8030080

**Published:** 2018-08-23

**Authors:** Yuhua Zhu, Xuemei Xian, Zhenzhen Wang, Yingchao Bi, Quangang Chen, Xufeng Han, Daoquan Tang, Renjin Chen

**Affiliations:** Laboratory Animal Center, Xuzhou Medical University, Xuzhou 221004, Jiangsu, China; zhuyuhua116@126.com (Y.Z.); xxuemay@163.com (X.X.); wangzhenzhen620@163.com (Z.W.); m15062145239@163.com (Y.B.); chenquangang@xzhmu.edu.cn (Q.C.); hxf229791518@163.com (X.H.); tangdq@xzhmu.edu.cn (D.T.)

**Keywords:** atherosclerosis, inflammation, anti-inflammatory therapy

## Abstract

Atherosclerosis is a chronic inflammatory disease; unstable atherosclerotic plaque rupture, vascular stenosis, or occlusion caused by platelet aggregation and thrombosis lead to acute cardiovascular disease. Atherosclerosis-related inflammation is mediated by proinflammatory cytokines, inflammatory signaling pathways, bioactive lipids, and adhesion molecules. This review discusses the effects of inflammation and the systemic inflammatory signaling pathway on atherosclerosis, the role of related signaling pathways in inflammation, the formation of atherosclerosis plaques, and the prospects of treating atherosclerosis by inhibiting inflammation.

## 1. Introduction

Atherosclerosis is a chronic cardiovascular disease that is harmful to human health; atherosclerosis is one of the most common causes of death in the elderly. The main lesions in atherosclerosis are characterized by lipid deposition in parts of the artery accompanied by smooth muscle cell and fibrous matrix proliferation, which gradually develop into the formation of an atherosclerotic plaque. Atherosclerosis is usually considered a chronic inflammatory disease [1] as inflammation plays an important role in all stages of the atherosclerotic process [2]. Inflammation acts as a common basis for the physiological and pathological changes throughout atherosclerosis initiation and development. Atherosclerosis is the pathological basis of cardiovascular disease while unstable atherosclerotic plaque rupture, platelet aggregation, and thrombosis cause stenosis or occlusion of blood vessels, leading to acute cardiovascular disease [3,4]. We introduce the effects of inflammation, markers, inflammatory signaling pathways, and anti-inflammatory treatments on atherosclerosis in this study, which will provide a new way to treat atherosclerosis for future research.

## 2. The Effects of Inflammation on Atherosclerosis Development

Inflammation has been proved to play an important role in the initiation and progression of atherosclerotic plaque. In early-stage atherosclerosis, the causes of atherosclerosis are endothelial injury, abnormal lipid metabolism, and hemodynamic damage; and the atherogenic process is considered to be accompanied by flow-mediated inflammatory changes in endothelial cells (ECs) [5]. When ECs are activated, they express monocyte chemoattractant protein-1, interleukin (IL)-8, intercellular adhesion molecule-1 (ICAM-1), vascular adhesion molecule-1 (VCAM-1), E-selectin, P-selectin, and other inflammatory factors, attracting lymphocytes and monocytes that bind to the endothelium and infiltrate arterial wall, and inflammation begin to happen [6]. Many cells and cytokines are involved in this process such as macrophages, lymphocytes (T and B cells), dendritic cells (DCs), ECs, vascular smooth muscle cells (VSMCs), ILs, adhesion molecules, and tumor necrosis factor (TNF-α) [7]. A large number of low-density lipoprotein (LDL) are modified to oxidized LDL (oxLDL), and accumulates in the vascular internal wall contributing to atheorosclerotic plaque development [6]. The monocytes differentiate to macrophages that engulf oxLDL deposits and transform into foam cells [8]. Proinflammatory monocytes that express high levels of Ly6C or Gr-1 preferentially accumulate in atherosclerotic plaques and adhere to cytokine-stimulated endothelial cells in vitro [9]. Other types of immune cells, such as DCs, T cells, B cells, and neutrophils participate in the intraplaque inflammation. Myeloid cells have attracted interest for altering the innate immunity response, studies in rabbit also showed evidence for myeloid cell proliferation in atherosclerotic lesions [10].

In late-stage atherosclerosis, a large number of macrophages and other inflammatory cytokines infiltrate the vessel wall, secrete matrix metalloproteinases (MMPs), and degrade collagen fibers in the extracellular matrix of the plaque, resulting in plaque rupture, bleeding, and thrombosis [11,12]. Infiltrating mast cells contribute to the proinflammatory milieu [13]. Upon activation, these cells release a host of mediators and enzymes, which may profoundly affect the atherosclerotic lesion.

The concerted action of all proinflammatory signals operating in the plaque not only enhances inflammation but also hampers renewal of the structural elements that support the mechanical stability of the inflamed tissue [14]. A variety of proinflammatory messengers are released by immune and vascular endothelial cells (VECs), activating cytokines, chemokines, bioactive lipid compounds, and adhesion molecules that maintain and enhance local inflammation and development of atherosclerotic lesions [6].

## 3. Markers in the Development of Atherosclerosis Inflammation

### 3.1. C-Reactive Protein 

The C-Reactive Protein (CRP) is mainly produced by IL-6, IL-1β, and TNFα stimulation in the liver. When tissue is damaged, the inflammatory response system is activated; macrophages accumulate in the damaged tissue and release large amounts of IL-6 and TNFα, which induce the liver to synthesize large amounts of CRP. Other tissues such as adipose tissue may be able to synthesize CRP under proinflammatory stimuli [15]. C-reactive protein can lead to vascular endothelial cell damage, preventing VECs from repairing and proliferating. The native circulating form of CRP is pentameric (pCRP), which is mostly released into the circulation after inflammatory stimuli [16]. As nonsoluble monomers CRP (mCRP), which are involved in the innate immune system by activating the complement cascade, in angiogenesis and in thrombosis [17,18,19]. C-reactive protein levels can often provide useful information for diagnosing, treating, and monitoring patients with atherosclerosis, and confirm patient responses to various stimulant factors [20]. Studies supporting a causal role point to evidence that CRP binds to LDL and is present in atherosclerotic plaques [21]. C-reactive protein is not present in the healthy vessel wall but it becomes detectable in the early stages of atherogenesis and accumulates during the progression of atherosclerosis [22]. C-reactive protein is considered a predictor of future cardiovascular events, and in the general population CRP levels are able to independently predict the risk of all-cause and cardiovascular mortality [23,24]. Therefore, CRP is a sensitivity index for inflammation, and the development and progression of atherosclerosis are directly linked to the risk of atherosclerosis indicators.

### 3.2. Interleukin-6

Interleukin-6 is a pleiotropic cytokine and involves innate and adaptive immunity system and serves to regulate the acute-phase response and chronic inflammation [25]. By measuring IL-6 levels in blood samples of coronary heart disease patients, it was found that blood IL-6 levels were higher in the coronary heart disease group than in healthy subjects. The more serious the disease, the higher the inflammatory index [26]. Interleukin-6 levels were significantly increased in cases of plaque rupture [27]. The genetic deficiency of IL-6 was found to enhance the formation of diet- and/or pathogen-associated atherosclerotic plaques [28]. Therefore, IL-6 may be a potential marker to predict the vulnerability of atherosclerotic plaques.

### 3.3. Adhesion Molecules

Adhesion molecules are proteins that mediate cell–cell and cell–extracellular matrix contact and adhesion. They are involved in cell recognition, activation, signal transduction, proliferation, differentiation, and metastasis by mediating interactions with corresponding ligands. They also mediate tissue inflammation, immune responses, and participate in and regulate thrombosis. Adhesion molecules play an important role in the development of atherosclerotic plaques [29]. The adhesion molecules associated with atherosclerosis are the selectin family, Immunoglobulin superfamily (IgSF), and integrin family.

The Selectin family are transmembrane glycoproteins, which mainly participate in the rolling adhesion of leukocytes. There are three types: endothelium-selectin (E-selectin), platelet-selectin (P-selectin), and leukocyte selectin (L-selectin). P-selectin plays an important role in the atherosclerotic process and is involved in the activation, rolling, and attachment of leukocytes, as well as the bonding of endothelial cells via ligand interaction [30]. Clinical studies have found that the level of P-selectin expression was positively correlated with the degree of atherosclerosis lesions and plaques. P-selectin is involved in the pathogenesis of acute cerebral infarction (ACI) patients, which is also an important indicator of ACI patients [31,32].

The IgSF have a molecular structure similar to immunoglobulins, which interact with integrin family adhesion molecules as receptors and ligands, and are involved in cell recognition and adhesion. There are three main types: ICAM-1, VCAM-1, and platelet endothelia cell adhesion molecule-1 (PECAM-1). Vascular cell adhesion molecule-1 and ICAM-1 are important members of the IgSF. Vascular cell adhesion molecule-1 (VLA-1) is a ligand of the VLA-4 (α4β1) integrin that is expressed on the surface of the ECs and is activated by cytokines; ICAM-1 is a ligand of LFA-1. High VCAM-1 and ICAM-1 expression promotes macrophage proliferation, resulting in a large number of macrophages accumulating in the plaque, therefore, increasing plaque instability [33]. Studies have found that VCAM-1 and ICAM-1 were closely related to angiogenesis [34]. In an atherosclerosis plaque, high VCAM-1 and ICAM-1 expression promotes neovascularization, which is characterized by mostly immature, highly permeable, brittle vessels. Intercellular adhesion molecule-1 expression is an early event in atherosclerotic lesions while VCAM-1 is probably expressed at a later stage [35].

Integrin family is a glycoprotein receptor widely found on many cell surfaces. There three main types: β1 subfamily, β2 subfamily, and β1 subfamily. As transmembrane receptors, integrins may integrate the internal actomyosin cytoskeleton with IgSF counter-receptors (VCAM-1 and ICAM-1) on adjacent cells [36]. Integrin signaling may affect multiple aspects of atherosclerosis, from the earliest induction of inflammation to the development of advanced fibrotic plaques. Integrin regulation of atherosclerotic plaque development and the suitability of integrins as potential therapeutic targets to limit cardiovascular disease [37].

### 3.4. Matrix Metalloproteinases

Matrix metalloproteinases (MMPs) are secreted by multiple cell types, including macrophages. Matrix metalloproteinases are thought to mediate the progression of stable atherosclerotic lesions to an unstable phenotype [38]. Matrix metalloproteinase-9 and MMP-2 are members of the MMP family that are closely related to the stability of atherosclerosis plaques. Matrix metalloproteinase-9, also known as gelatinase B, is mainly produced by activated macrophages and VSMCs. Matrix metalloproteinase-9 also plays a critical role in the progression of atherosclerosis, loss of MMP-9 reduced the atherosclerotic burden throughout the aorta and impaired macrophage infiltration and collagen deposition [39]. Clinical studies have shown that MMP-9 levels in atherosclerosis vulnerable plaques were higher than in the nonvulnerable plaque and normal control groups, and that MMP-9 and atherosclerosis plaque vulnerability were positively correlated [40]. The results of animal experiments showed that the level of MMP-9 expression in the arterial and peripheral vessels of the atherosclerosis animal model was significantly higher, and the level of the elevation was positively correlated with the degree of atherosclerosis lesion; therefore, MMP-9 is one of the most important biological indexes for predicting plaque vulnerability in atherosclerosis [41,42]. Matrix metalloproteinase-2 is an independent risk factor for vascular remodeling and is one of the key enzymes involved in the degradation mechanism. When the atherosclerosis endothelium is damaged, monocytes/macrophages and ECs secrete MMP-2. Matrix metalloproteinase2 can promote extracellular matrix degradation and promote the proliferation and migration of VSMCs after plaque formation [43]. At the early stage of atherosclerosis, activity of MMP-2 can degrade the basal membrane of ECs, and decrease the defense of ECs, and further to lead to LDL to tunica intima. Matrix metalloproteinase2 deficiency reduces the atherosclerotic plaque lesion formation in apolipoprotein E-deficient (apoE^−/−^) mice; MMP-2 may induce plaque stability by accumulating SMC [44,45]. Moreover, MMP-2 and MMP-9 have been shown critical contributions in human rheumatoid synovium, intratumor angiogenesis, invasion of tumor progression, and so on [46]. Other MMPs members (MMP-12 and MMP-13) have also been studied; MMP-12 deficiency reduced plaque size and MMP-13 deficiency had no effect on plaque development but decreased collagen content in the plaque in apoE^−/−^ mice [47,48].

## 4. Atherosclerosis Related Inflammatory Signaling Pathways

### 4.1. Toll-Like Receptor 4 Signaling

Toll-like receptor 4 (TLR4), a typical representative of pattern recognition receptors in innate immune responses, which can activate the transcription factors nuclear factor-κB (NF-κB) leading to the production of proinflammatory cytokines [49]. Toll-like receptor 4 mainly regulates *ABCG1*, which is a key gene that mediates inflammation and cellular lipid accumulation [50]. Toll-like receptor 4 induces inflammation and lipid accumulation in VSMCs by downregulating *ABCG1* expression through the Peroxisome proliferator activated receptor gamma (PPAR γ)/liver X receptor alpha (LXRα) signaling pathway [51]. Toll-like receptor 4 signaling in atherosclerosis plays an important role in activation of inflammation in activation of inflammation and lipid accumulation, which are all associated with atherosclerosis plaque progression and vulnerability [52]. In human aortic SMC, LPS stimulates TLR4 signaling to promote the release of MCP-1, IL-1α, and IL-6 [53]. Contribution of TLR4 signaling in intermittent hypoxia-mediated atherosclerosis progression [54]. So TLR4 signaling maybe an ideal target for interfering in the AS progression.

### 4.2. Nuclear Factor-κB Signaling

The NF-κB family of transcription factors has an essential role in inflammation and innate immunity. Activation of the NF-κB pathway plays a central role in inflammation and can be induced by gene encoding, proinflammatory cytokines, adhesion molecules, chemokines, growth factors, and monocytes bound to the endothelium [55]; Nuclear factor-κB transcription factors are key regulators of inflammation and cell death in the pathogenesis of atherosclerosis [56]. After low density lipoprotein receptor (LDLR)^−/−^ mice were fed a high-fat diet over an extended duration, the endothelium showed enhanced NF-κB activity, and the chance of local proximal aortic atherosclerosis increased [57]. However, inhibition of NF-κB activation in macrophages causes a reduction of foam cell formation, anti-apoptosis, and anti-inflammation [58]. Triggering the activation of TLR4/NF-κB signaling and the downstream proinflammatory responses promotes the plaque growth and instability [54].

### 4.3. Janus Kinase (JAK)-Signal Transducer and Activator of Transcription (STAT) Signaling

Janus kinase (JAK)-signal transducer and activator of transcription (STAT) is an important signaling pathway regulating the initiation/progression of atherosclerosis [59]. The JAK-STAT pathway was activated by cytokines by the JAK kinases (JAK1, JAK2, JAK3), and tyrosine kinase (Tyk) 2, which was also found in atherosclerotic lesions [60]. In atherosclerosis model mice, the IL-6 and TNF-α level were significantly increased in plasma and aortic tissues when p-STAT3 levels were increased [61]. In terms of immunoregulation, STAT4 and STAT6 are essential for cellular differentiation. IL-4 activates STAT6 to promote the differentiation of T helper (Th) 2 cells, and Th2 has anti-atherosclerosis activity [62]. Interleukin-12 activates STAT4, which drives the initial differentiation of T-cells into Th 1, which secretes interferon (IFN)-γ. In atherosclerosis, Th cells respond to the Th1 type, which secretes large amounts of IFN-γ and TNF-α, mediating macrophage activation, and promoting atherosclerosis development and plaque enlargement [63]. Sustaining STAT1/STAT3 activation and aggravates lesion development [64].

## 5. Anti-Inflammatory Treatments for Atherosclerosis

### 5.1. HMG-CoA Reductase Inhibitors

Statins are mainly used to specifically reduce cholesterol synthesis; however, clinical studies have also shown that statins can effectively reduce the level of inflammatory biomarkers such as CRP, independent of reduced cholesterol levels [65]. The anti-inflammatory effect of statins may be partly through its lipid lowering effects, but there is substantial evidence that statins have direct anti-inflammatory effects on cells involved in the development and rupture of atherosclerotic plaques [66]. In ECs, statins can reduce VCAM-1 and ICAM-1 expression and inhibit the capture of monocytes. In vivo experiments have shown that statins can reduce macrophage growth and their MMP activity, which can stabilize atherosclerosis vulnerable plaques [67].

The molecular target of all statins is 3-Hydroxymethyl-3-glutaryl-CoA (HMG-CoA) reductase, which is responsible for the initial and rate limiting step of cholesterol synthesis. The HMG-CoA reductase inhibitors are potent inhibitors of cholesterol biosynthesis by blocking the hepatic conversion of HMG-CoA to l-mevalonate in the cholesterol biosynthetic pathway, and finally decreasing serum cholesterol level [68]. HMG-CoA inhibitors display pleiotropic effects in anti-inflammatory and antiproliferative activities [69]. Polymeric micelles (PM) are clinically applicable nanomedicines targeting HMG-CoA reductase, which reduce the macrophage burden in advanced atherosclerotic plaques in comparison to high-density lipoprotein (HDL) and liposomes [70]; so, HMG-CoA inhibitors play an important role in treating atherosclerosis.

### 5.2. Phospholipase A2 Inhibitors

Members of the phospholipase A2 (PLA2) superfamily are associated with lipoproteins and modify phospholipids in LDL particles to promote atherosclerosis development. Lipoprotein-associated phospholipase A2 (lpPLA2) plays prominent pro-atherogenic and proinflammatory roles. Secretory PLA2 (sPLA2) is released by the smooth muscle cells and hepatocytes in the acute phase of inflammation [6]. Phospholipase A2 inhibitors could be potentially useful for atherosclerosis therapy. Darapladib is an lpPLA2 inhibitor, which showed a great efficacy in reducing lpPLA2 activity by 95%, and further, demonstrated anti-atherosclerotic and anti-inflammatory activities in diabetic and hypercholesterolemic pigs [71].

Varespladib is an inhibitor of sPLA, which can provide more beneficial effects including significant reduction of LDL-C and inflammatory markers in patients with acute coronary syndrome [72]. However, some inhibitors of PLA2 failed to reduce the risk of major atherosclerosis events in some clinical trials, event to produce some multiple adverse side effects. Therefore, PLA2 inhibitors need to be studied in clinical trials [73].

### 5.3. Glucagon-Like Peptide-1 

Type 2 diabetes has a high probability of cardiovascular disease, and hyperglycemia is a key factor in the development of cardiovascular disease. Furthermore, inflammation is an important pathologic factor in diabetic heart disease. Glucagon-like peptide-1 (GLP-1), translated from the glucagon gene product, is proteolytically cleaved, and mainly secreted from L cells of the distal gut and used to ingest nutrients.

Glucagon-like peptide-1 contributes to glucose homeostasis by stimulating insulin secretion in pancreatic β cells, and also exerts cardioprotective and vasodilatory actions [74,75]. Glucagon-like peptide-1 can suppress monocyte/macrophage infiltration into the aortic wall and the development of atherosclerotic lesions in apoE^−/−^ mice. Glucagon-like peptide-1 with its receptor (GLP-1R) suppress exudate peritoneal macrophage foam cell formation induced by oxLDL [76]. A GLP-1 analog liraglutide suppresses macrophage foam cell formation, to prevent the development of atherosclerotic lesions [77]. Thus, GLP-1 could be a potential therapeutic target on atherosclerosis.

### 5.4. Inhibition of Inflammatory Cytokines

Proinflammatory cytokines play an important role in atherosclerosis-related inflammation, they include IL-1β, IL-6, TNF-α, IFN-γ, and 5-lipoxygenase (5-LO). So suppressing production of these cytokines in VECs may be of therapeutic importance for anti-inflammatory treatment of atherosclerosis. IL-1β can be produced by activated macrophages, neutrophils, and ECs. Expression of IL-1β is elevated in atherosclerotic lesions as compared to unaffected tissue, and IL-1β can be seen a target of clinical potential for anti-inflammatory treatment of atherosclerosis [78]. Interleukin-1β-specific monoclonal antibody protects against cardiovascular risks [79]. Interleukin-6 is produced by macrophages and T cells during the early stage of the immune response and inflammation, and has proinflammatory properties [80]. Tocilizumab, an IL-6 receptor-specific humanized monoclonal antibody, is used to inhibit IL-6-dependent signaling. Tocilizumab displays beneficial effects on surrogate markers of vascular risk by reducing levels of sPLA2 [81]. Several TNF-α blockers are available for treatment of inflammatory disorders. Several TNF-α-specific monoclonal antibodies and TNF-α inhibitors were used to suppress atherosclerosis development [82,83]. However, some adverse effects of these were described, and more studies are needed to translate these findings into clinical practice. Interferon-γ can activate macrophages leading to increased proinflammatory signaling, lipid uptake, and foam cell formation. The role of IFN-γ is well described in atherosclerotic plaque progression [84]. Anti-IFN-γ therapies are hindered by the associated adverse events for treatment of cardiovascular diseases, but downstream components of the IFN-γ signaling pathway can be exploited for developing antiatherosclerosis therapies. 5-lipoxygenase (5-LO) has emerged as a potential therapeutic target for reduction of plaque inflammation [85]. Inhibition of 5-LO attenuates plaque progression in atherosclerosis mice models [86]. Inhibition of 5-LO reduces neointimal thickening and macrophage infiltration in hypercholesterolemic rabbits [87]. Inhibition of 5-LO may be a potential therapeutic target on atherosclerosis.

### 5.5. Peroxisome Proliferator-Activated Receptors

Peroxisome proliferator-activated receptors, as fatty acids sensors, have been therapeutic targets in several human lipid metabolic diseases, such as obesity, atherosclerosis, diabetes, hyperlipidemia, and nonalcoholic fatty liver disease. There are three types of PPARs which have been identified: PPARα, PPARβ/δ, and PPARγ. Peroxisome proliferator activated receptors have some common ground in their anti-atherosclerotic effects. PPARα and PPARγ both prevent foam cell formation and atherosclerosis development in ApoE^−/−^ and LDLR^−/−^ mice [88]. Peroxisome proliferator activated receptors have been a critical interface for inflammation and cholesterol homeostasis. Peroxisome proliferator activated receptors may be a potential therapeutic target on atherosclerosis.

## 6. Summary

Atherosclerosis is a chronic inflammatory disease, and it is generally considered that a variety of inflammatory cells and inflammatory factors play significant roles throughout its pathogenesis. These responses include abnormal responses to a variety of damages to the vascular wall, which induce classic inflammation to degeneration, exudation, and hyperplasia. The detection of inflammatory biomarkers such as CRP and adhesion molecules IL-6 and MMPs can be a good means of diagnosing atherosclerosis and cardiovascular disease. Toll-like receptor 4, NF-κB, and JAK/STAT are atherosclerosis-related inflammatory signaling pathways. By studying these signaling pathways, we have gained a better understanding of the pathogenesis of atherosclerosis. The roles of anti-inflammatory treatments and inhibition of inflammatory cytokines are currently well-known. Therefore, it is worth discussing and studying how to prevent the occurrence and development of atherosclerosis through anti-inflammatory and immunological regulation with the aim of treating the various risk factors associated with atherosclerosis.
